# The Effect of Denatured Albumin with Concentrated Growth Factors in Minimally Invasive Sinus Piezosurgery: Preliminary Pilot Study Results

**DOI:** 10.1055/s-0043-1772250

**Published:** 2023-09-20

**Authors:** Vasileios Ntontoulos, Nikolaos Dabarakis

**Affiliations:** 1 Department of Dentoalveolar Surgery, Surgical Implantology & Roentgenology, Aristotle University of Thessaloniki, Greece

**Keywords:** bioengineering, sinus floor augmentations, Piezosurgery, serum albumin, dental implants

## Abstract

A new protocol with albumin-concentrated growth factor (CGF) is investigated through Piezosurgery as a minimally invasive alternative to sinus-floor-augmentation that is associated with high morbidity and high incidence of sinusitis. The clinical sample consists of five patients (three men and two women) with an average age of 53.75 ± 3.59 years and a mean height of 3.7 ± 1.22 mm of residual bone. The Piezo-Alb-CGF protocol consists of a minimally invasive transcrestal approach with or without flap, piezosurgery preparation, applying the Schneiderian membrane's hydrodynamic detachment-elevation, injecting albumin-CGF into the sinus, optional bone grafting and implantation, and evaluation for 2 to 6 months postoperatively. Eight implants were placed without complications. After 4 to 6 months, cone-beam computed tomography and panoramic radiographs showed total osseointegration and the formation of new bone. In addition, a year of clinical follow-up was performed. There was a positive correlation between implant stability quotient values at all protocol stages. The significance level was 5%. Albumin-CGF regenerative protocol promotes new bone formation, reduces postoperative morbidity, and shortens healing time. It also offers a uniform and safe hydraulic membrane lift and bicortical implant fixation, even in cases with a residual bone height below 6 mm.

## Introduction

The maxillary sinus floor augmentation has been a reliable implant treatment protocol 1 for the posterior maxilla, especially in severe resorption, bone atrophy, and sinus pneumatization. 2 However, the high incidence of Schneider's membrane perforation and postoperative sinusitis has shown that the direct sinus augmentation technique (SAT) can be considered a highly morbid invasive approach. In transcrestal closed-type cases with indirect compression osteotomy (Summers, 1994-indirect SAT), the method is considered not invasive and relatively safe. 3 Many protocols and techniques have been introduced in dental practice based on the same biological principles 4 5 and, among others, the Intralift technique with Piezotome and hydrostatic lifting of the membrane. 6 Bioengineering and regenerative medicine collaborations have provided new blood-derived autologous grafts, such as platelet-rich plasma (PRP), platelet-rich fibrin (PRF), and concentrated growth factors (CGF). 7 The clinical use of these platelet transforming growth factors (transforming growth factor-b1 [TGF-b1] and vascular endothelial growth factor [VEGF]) involved in fibrin matrix-CGF is considered a new, promising, minimally invasive, reliable, and alternative proposition in combination with traditional bone augmentative techniques. 8

In extreme cases with residual bone height (RBH) below 6.0 mm, to avoid an open lateral window approach, the innovative Piezo-Alb-CGF (PAC) 9 clinical protocol could allow alternatively the placement of dental implants in the sinus area using a closed-type crestal approach.

The purpose of this study is to demonstrate that the healing and osseoregenerative properties of denatured albumin (with or without bone grafts) used within this Alb+CGF protocol can function simultaneously: (a) as a space filler, (b) as a healing-sealing agent in a possible membrane perforation, and (c) as an inductive bone substitute (which also compensates for sinus pneumatization).

## Materials and Methods

The present clinical pilot study was conducted in the Department of Dentoalveolar Surgery, Surgical Implantology, and Roentgenology, Aristotle University of Thessaloniki, Greece, from July 2021 to September 2022. The study included five healthy subjects (three men and two women) with a mean age of 53.75 ± 3,59 years ranging from 49 to 63 years. The inclusion criteria were posterior maxillary edentulism, opting for prosthetic restoration with dental implants with the need for a maxillary sinus lift. The study protocol followed the Helsinki Declaration, guidelines had been approved by the University Ethical Committee/Dental School, Aristotle University of Thessaloniki-Greece (Protocol Number: 125/13-07-2021), and written informed consent was obtained from each subject before the procedure.

The RBH was determined by the distance between the crest of the alveolar ridge and the cortical sinus floor (in the CBCT sagittal view), while the residual bone width (RBW) was measured by the horizontal line of the distance between the buccal and palatal cortical, 2 mm above the crest (in the coronal view). 10

RBH ranged from 2.1 to 6.0 mm, averaging 3.7 ± 1.22 mm. Previous sinus pathologies were evaluated according to the radiological classification of class I to class IV of Di Girolamo et al on the thickness and pathology of the Schneiderian membrane. 11

A total of eight implants (AnyRidge; MegaGen, Seoul, S.Korea) with diameters Ø 4.0 / 4.5 mm and lengths of 10 and 11.5 mm were placed simultaneously with the PAC protocol, using a circular tissue punch without a flap (flapless technique).

**Fig. 1:**
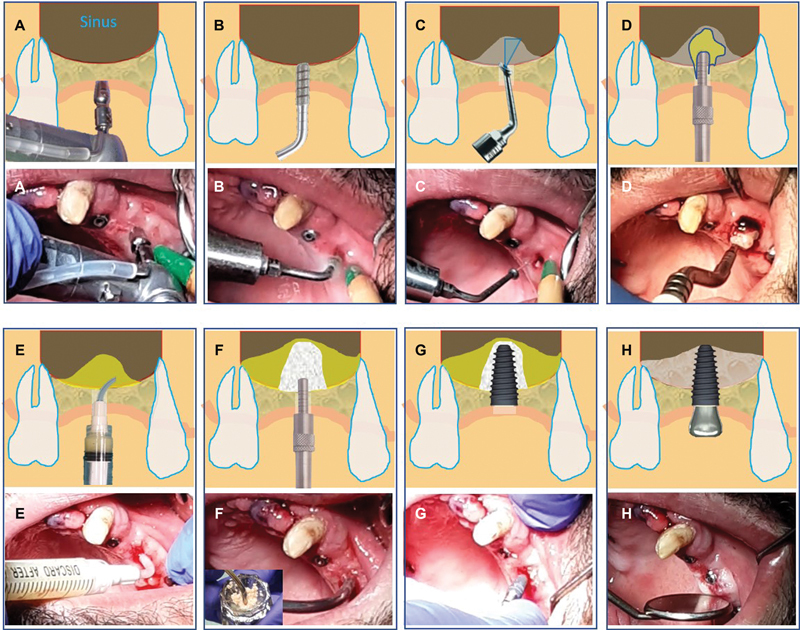
Up: Piezo-Alb-CGF (PAC) protocol schematic illustration, down: PAC protocol clinical sequence (surgery). ( **A** ) Tissue-punch flapless approach, ( **B** ) Piezosurgery preparation by TKW1-TKW4 tips, ( **C** ) membrane cavitation by TKW5 tip, ( **D** ) concentrated growth factor (CGF)-matrix into the sinus cavity below the membrane, (E) albumin + CGF gel injection, ( **F** ) bone grafting, ( **G** ) implantation, ( **H** ) healing screw, follow-up 4 to 6 months postoperatively, new bone formation below the membrane.

This novel clinical transcrestal PAC protocol (Piezo-Alb-CGF) consisted of the following phases ( Fig. 1 ).

**Phase 1: Surgical approach** ( Fig. 1A ) **and Piezosurgery preparation** ( Fig. 1B )

Piezosurgery was performed without a flap using a circular tissue punch and Piezotome CUBE (Satelec SAS, Acteon Group, Mérignac, France), a Piezosurgical appliance using non-rotary diamond tips (TKW1- TKW4) for osteotomies. 12

**Phase 2: Sinus membrane cavitation** ( Fig. 1C )

Subsequently, the initial microcavitation and detachment of the sinus membrane were performed by the TKW5 tip in the shape of a “trumpet” (Intralift Kit; Satelec SAS, Acteon Group, Mérignac, France).

**Phase 3: Insertion of the CGF matrix into the sinus** ( Fig. 1D )

**Fig. 2:**
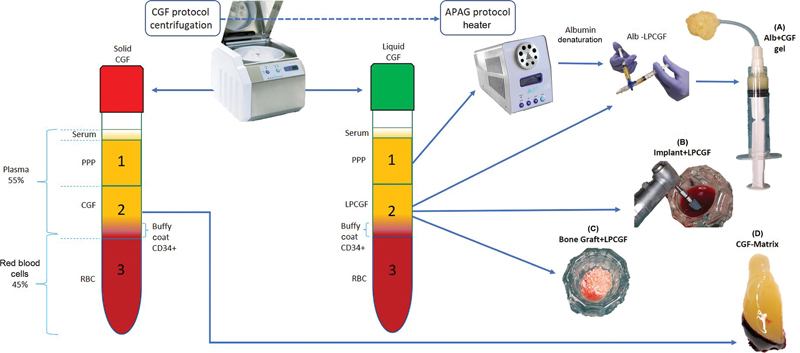
Blood derivatives management in the Piezo-Alb-CGF protocol: ( **A** ) albumin + concentrated growth factor (Alb + CGF) gel, ( **B** ) implant + liquid-phase CGF (Implant + LPCGF), ( **C** ) Bone Graft + LPCGF, and ( **D** ) Matrix CGF.

A Valsalva test was performed to detect any possible perforation. The autologous biological grafts employed in the presented protocol were based on the CGF protocol 13 ( Fig. 2 )

The CGF protocol involves blood sampling and centrifugation with the Medifuge MF200 device (Silfradent srl, ForlÌ-Cesena, Italy) in 9 mL sterile test tubes (red and green). Before the injection of Alb + CGF gel, one or two CGF matrices membranes (red) ( Fig. 2D ) were inserted as a safety layer to compensate for any possible membrane micro perforation.

**Phase 4: Alb** **+** **CGF gel injection within the sinus** ( Fig. 1E )

This gel, which is denatured human serum albumin 9 from the APAG (activated plasma albumin gel) protocol, was mixed with autologous CGF as an autologous osteoinductive and slow absorption graft (for at least 6 to 8 weeks) 14 and was inserted below the membrane as a scaffold ( Fig. 2A ). The APAG protocol involves centrifugation (green tubes) of three blood fractions to select the upper part, which is then sucked with a syringe and placed in the APAG heater (Silfradent srl, ForlÌ-Cesena, Italy). After 10 minutes at a temperature of 75°C, denatured albumin or APAG polymeric gel is left to cool for 10 minutes at room temperature in a dark area before mixing with the liquid-phase CGF (LP-CGF) of the middle fraction (green) in a second syringe, the CGF and CD34+ hematopoietic stem cells 15 using a common three-way mixer component (female-female luer lock connector). 14

**Phase 5: Sinus bone grafting** ( Fig. 1F ) **and implantation** ( Fig. 1G )

Simultaneous implant placement after immersion in LP-CGF (green) to enrich the implant surface with growth factors ( Fig. 2B ) and also placing a mixture of LP-CGF with bone graft ( Fig. 2C ) to achieve better primary stability.

**Phase 6: Wound closure and 4 to 6 months postoperative evaluation** ( Fig. 1H )

The postoperative instructions included an ice pack (2 hours) antibiotics (amoxycillin 875 mg + clavulanic acid 125 mg per os two times /day for 5–7 days), nonsteroidal anti-inflammatory drugs, and nasal decongestants if needed. In this study, all cases were performed using the flapless technique, and healing screws were placed on the implants shortly after surgery.

Radiological evaluation was performed in all cases with cone-beam computed tomography after surgery (4-6 months), where the gain in RBH was measured. The clinical evaluation of implant stability was performed using resonance frequency analysis with implant stability quotient (ISQ, Osstell-Sweden). 16 ISQ values were recorded at implant placement, 2 months later, and then each month until the final prosthetic restoration. The healing period (for implant osseointegration) was defined as the required time (in months) for the ISQ value (initial to final) to be stabilized more than 65 upwards (ISQ alteration) after surgery. All implants were loaded 5 to 6 months after placement, showing medium to high stability, according to the ISQ system (x/y, x = lingual buccal direction and y = distal mesial direction, and their mean ISQ value was defined as x + y/2) ( Table 1A ).

**Table 1:** Statistical results of the eight implants: A. All variables and their mean ± SD, B. T-test for Pearson correlation (α = 0.05). There were three positive correlations ( *p* -value < 0.05) between initial and final ISQ (correlation: 0.912; *p* = 0.002), initial and restoration ISQ (correlation: 0.763; *p* = 0.028), final and restoration ISQ (correlation: 0.956; *p* = 0.0002), and a negative correlation (p < 0.05) between initial ISQ and ISQ alteration (correlation: −0.841; *p* = 0.009)

Patients no	Gender M/F	Implant site	Age (y)	RBW (mm)	RBH (mm)	Gain/RBH (mm)	Healing (mo)	Initial ISQ	Final ISQ	Alteration ISQ	Restoration (mo)	Restoration ISQ
1	F	#27	63	10.0	3.0	7.0	2	60.5	72.5	12.0	6	77.0
2	M	#27	57	10.2	3.3	7.7	3	74.5	79.5	5.0	6	81.5
3	M	#26	49	10.0	2.1	8.9	4	70.0	72.0	2.0	5	72.0
4	M	#25	53	6.0	5.0	10.0	5	76.0	78.5	2.5	6	80.0
		#26		7..2	3.2	6.5	5	68.5	72.0	3.5	6	72.0
5	F	#15	56	7.6	6.0	3.4	3	76.5	80.0	3.5	5	81.0
		#16		13.9	3.4	6.6	3	68.5	74.5	6.0	5	75.0
		#17		13.3	3.6	6.4	4	55.0	65.5	10.5	5	67.5
**Mean** ± SD*			**53.75** ** ± 3.59**	**9.77** ** ± 2.81**	**3.7** ** ± 1.22**	**7.06** ** ± 1.95**	**3.62** ** ± 1.06**	**68.68** ** ± 7.60**	**74.57** ** ± 5.23**	**5.62** ** ± 3.72**	**5.5** ** ± 0.53**	**75.75** ** ± 5.02**
**A** **B** *. Abbreviation: SD, standard deviation. **Correlation is significant at the 0.05 level (2-tailed). ***Correlation is significant at the 0.01 level (2-tailed).												
			**Pearson correlation**	RBW (mm)	RBH (mm)	Gain/RBH (mm)	Healing (mo)	Initial ISQ	Final ISQ	Alteration ISQ	Restoration (mo)	Restoration ISQ
			RBW	1								
			RBH	−0.45134	1							
			Gain/RBH	−0.13627	−0.43257	1						
			Healing	−0.43952	0.0658576	0.4121573	1					
			Initial ISQ	−0.61638	0.4640661	0.0841043	0.178295	1				
			Final ISQ	−0.53067	0.555310	−0.034323	−0.15272	0.911868***	1			
			Alteration	0.559646	−0.215935	−0.217115	−0.5657	−0.84082***	−0.54451	1		
			Restor/mo	−0.54183	−0.065341	0.4029487	0.125988	0.167008	0.286012	0.0359210	1	
			Restor/ISQ	−0.48602	0.5406049	−0.030854	−0.32811	0.762899**	0.956239***	−0.2978325	0.3986205	1

Postoperative complications were also recorded. All patients were monitored for 1 year after prosthetic rehabilitation.

Statistical analysis was performed with SPSS (V.25; IBM, Chicago, Illinois, United States). The means were compared using the paired-sample t-test and the correlation of variables using Pearson's correlation. The significance level was set at α =0.05 ( Table 1B ).

## Results

The study included five healthy subjects (three men and two women) with a mean age of 53.75 ± 3.59 years ranging from 49 to 63 years. None of the patients in this study had serious complications (intraoperatively and postoperatively).

All implants were osseointegrated and loaded 2 to 6 months after placement, showing sufficient to high stability (ISQ-Osstell) until their final restoration ( Table 1A ) and were also clinically stable and functional at the annual prosthetic follow-up (survival rate: 100%).

**Fig. 3:**
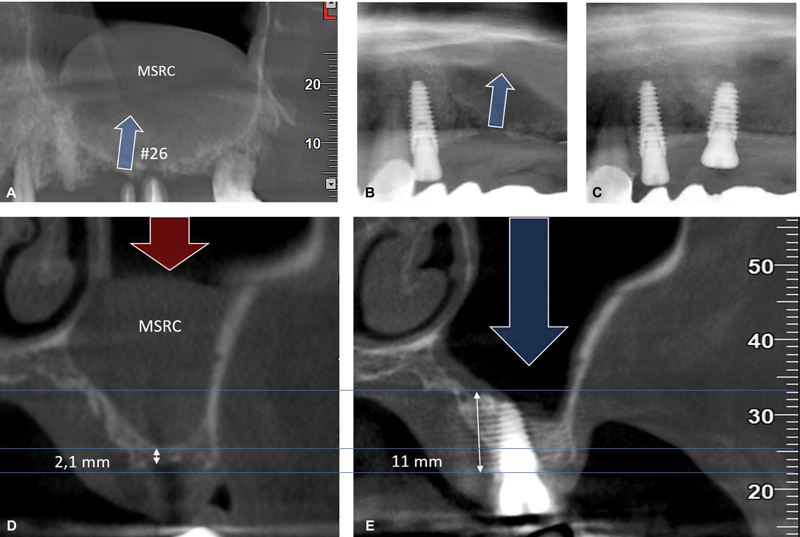
Sinus pathology—asymptomatic maxillary sinus retention cyst (MSRC). Sagittal plane: ( **A** ) before root extraction no 26, ( **B** ) after extraction with only concentrated growth factor (CGF)-matrix application to oroantral communication, and ( **C** ) just after Piezo-Alb-CGF (PAC) protocol. Coronal plane: ( **D** ) pre-op exam, ( **E** ) MSRC healed after PAC—4 months, bone augmentation (from 2.1 to 11mm), and implant no 26 (function as “tent pole”) osseointegration—implant stability quotient = 70/74.

All diagnostic images (6 months after surgery) showed complete implant osseointegration and new bone formation ( Figs. 3 4 5) (with a minimum to zero projection into the antral cavity always under the membrane). In all cases, there was no bone marginal loss, but a gain ranging from 3.4 to 10.0 mm with a mean gain in RBH of 7.06 ± 1.95 mm, without sinus infection or other pathology, even in the case of an asymptomatic maxillary sinus retention cyst, which was healed in two stages after the application of the PAC protocol ( Fig. 3 ).

**Fig. 4:**
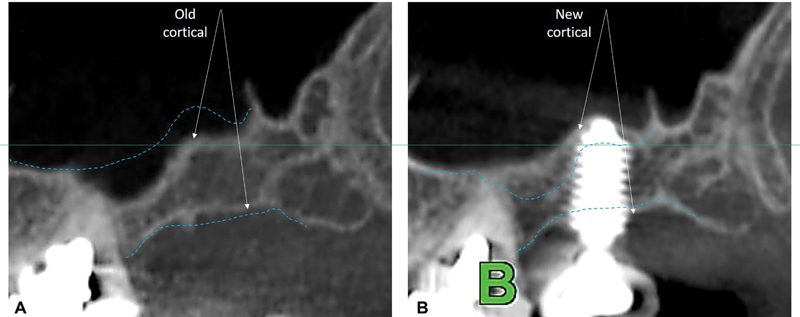
Uniform-safe hydraulic membrane lift—before ( **A** ) and after 12 months ( **B** ) due to albumin's gel-like texture without a graft. Note new sinus floor cortical formation and bicortical implant fixation.

**Fig. 5:**
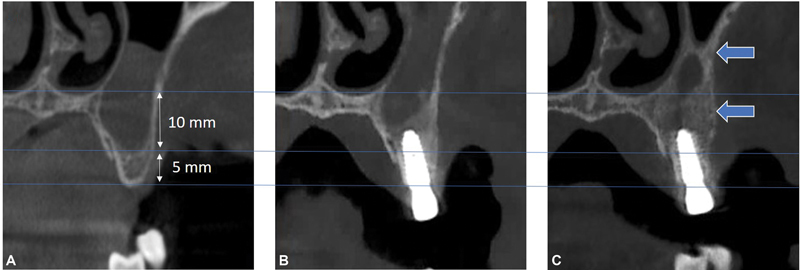
Piezo-Alb-CGF protocol with fully albumin- concentrated growth factor without bone graft: ( **A** ) preoperative, ( **B** ) bone remodeling 5 months postoperatively, ( **C** ) and impressive bone augmentation with natural texture, 10 months postoperatively.

## Discussion

The autologous graft of Alb + CGF gel used in this study was free of allergic reaction and integrated without complication. It was rich in regenerative factors and showed improved biomechanical and biochemical properties, such as slow absorption, stability, and plasticity, 15 making it suitable for any regenerative process in hard and soft tissues. 17

This protocol minimizes perioperative bleeding (avoiding posterior superior alveolar artery), morbidity, and patient discomfort while providing better visibility during surgery.

The use of Piezotomy for only hard tissue surgeries ensures maximum implant primary stability 12 while achieving bicortical fixation ( Fig. 4 ) even at a RBH of less than 6 mm and reducing the risk of membrane perforation due to its hydrodynamic lift ( Fig. 1C ).

There is a similar perspective in the clinical study by Alhayati and Al-Anee with implant placement by the osseous densification of the RBH of more than or equal to 2.0 less than 6.0 mm after 6 months of the osseous healing period, and this also showed higher primary and secondary implant stability postoperatively. 18

In only three difficult clinical situations that require sinus augmentation with 1.5 mm RBH (0.4–1.5 mm), in the study by Salgar with osseodensification, all healing was rapid and uneventful, without instances of sinus membrane perforation and vertical increase in sinus bone height ranges from 10.3 to 13.6 mm. 19 Although the gain in the minimum RBH for these studies, the ultimate risk of membrane perforation remains unsolved.

Instead, the top priority of this proposed method is to protect the membrane due to the gelatinous state in which albumin is injected into the sinus cavity by lifting the membrane hydraulically and horizontally to its maximum surface area. This is also superior to the open lateral technique of mechanical detachment (with sharp tools), having in parallel the bicortical fixation of the implant.

The sinus grafting process usually involves biomaterials under an intact sinus membrane to increase the bone volume. On the other hand, any possible membrane perforation can lead to undesirable consequences. This can be avoided by using autologous growth factors (TGF-b1 and VEGF) 7 derived from the platelets that are responsible for the healing of perforation, rupture, or soft tissue injury ( Figs. 3-5 ).

An implant of soft bone design (D3–D4) that has deep cutting threads to achieve initial primary stability can be placed with generally medium to high ISQ values (55-75) ( Table 1A ); it also plays the role of a tent pole ( Fig. 3E ), 20 against sinus pneumatization ( Figs. 3 4 5 ).

Regarding the integration of denatured albumin, in this study, it has also been observed that the more homogeneous and semifluid composition of the grafting material surrounding the implant, the better and more natural the integration of an implant into the recipient site ( Fig. 5 ).

All the above seem to underline the Alb-CGF potential as a sinus cavity graft due to (a) its healing-sealing properties ( Fig. 3B ), (b) its uniform Schneiderian membrane elevation, (c) its contribution to the formation of a new sinus cortical ( Fig. 4 ), (d) its ossification with a texture similar to the native bone ( Fig. 5 ), and (e) its viscosity and therefore of its excellent bone-to-graft contact, and graft-to-implant contact ( Figs. 3 4 5 ).

The preliminary promising results of this study introducing the innovative clinical use of denatured albumin with autologous platelet transforming growth factors (TGF-b1 and VEGF) and stem cells CD34+ (CGF) require further research and investigation to improve the biomechanical and biochemical properties of autologous growth factors and platelet concentrations in order to validate this protocol.

## Conclusion

The new complete minimally invasive protocol-PAC (denatured albumin with CGF) in sinus transcrestal piezosurgery with or without bone grafts appears to function simultaneously as a space filler, a healing-sealing agent in a possible membrane perforation just like in an oroantral communication in patient no 3 ( Fig. 3B ), and an inductive bone substitute while compensating for sinus pneumatization. The latter allows albumin to function as the ideal biological scaffold infiltrated by osteoblasts due to its proximity to bone structures up to its complete ossification (4–6 months).

The unique regenerative properties of alb-CGF in cases with a RBH below 6 mm reduce the healing period (for implant osseointegration) and minimize postoperative morbidity while promoting new bone formation.
